# Impedance spectroscopy of changes in skin-electrode impedance induced by motion

**DOI:** 10.1186/1475-925X-13-149

**Published:** 2014-11-18

**Authors:** Alper Cömert, Jari Hyttinen

**Affiliations:** Department of Electronics and Communications Engineering, Tampere University of Technology, BioMediTech, Tampere Finland

**Keywords:** Skin-electrode impedance, Motion artifact, Surface electrodes, Textile electrodes, ECG, EMG

## Abstract

**Background:**

The motion artifact is an ever-present challenge in the mobile monitoring of surface potentials. Skin-electrode impedance is investigated as an input parameter to detect the motion artifact and to reduce it using various methods. However, the impact of the used impedance measurement frequency on the relationship between measured impedance and the motion artifact and the relationship between the impedance and the motion is not well understood.

**Methods:**

In this paper, for the first time, we present the simultaneous measurement of impedance at 8 current frequencies during the application of controlled motion to the electrode at monitored electrode mounting force. Three interwoven frequency groupings are used to obtain a spectrum of 24 frequencies between 25 Hz and 1 MHz for ten volunteers. Consequently, the surface potential and one channel of ECG are measured from the electrode subject to controlled motion. The signals are then analyzed in time and frequency domain.

**Results:**

The results show that the different frequencies of impedance measurements do not reflect the motion in the same manner. The best correlation between impedance and the applied motion was seen at impedance current frequencies above 17 kHz. For resistance this relationship existed for frequencies above 11 kHz, Reactance did not show good time domain correlation, but had good frequency domain correlation at frequencies higher than 42 kHz. Overall, we found that the impedance signal correlated well with the applied motion; however impedance had lower correlation to actual motion artifact signal.

**Conclusion:**

Based on our results, we can conclude that the current frequency used for the impedance measurement has a great effect on the relationship of the measurement to the applied motion and its relationship with the resulting motion artifact. Therefore, when flat textile contact biopotential electrodes are used, frequencies higher than 17 kHz are best suited for impedance measurements intended for the estimation of electrode motion or motion artifact. For resistance, the best frequencies to use are higher than 11 kHz.

## Introduction

With the reduction in the size and power consumption of electronics, the market and the applications for biosignal monitoring systems such as mobile electrocardiogram (EKG) or mobile electromyography (EMG) are increasing. These systems are designed for use in everyday activities as well as for long-term monitoring.

Wearable applications that are used for the monitoring of surface biopotentials either long-term or during everyday activities in non-medical settings have their own specific problems that do not exist to the same extent in more conventional applications in controlled environments. The long-term stability of the electrodes and the motion artifact are two of the more prevalent problems. Depending on the application, one or both of these problems can be the dominant factor that determines the reliability of the system.

Many methods have been proposed to deal with the motion issue. In earlier studies on the motion issue, several methods such as adaptive filtering have been used to predict the motion artifact. These methods include optical sensors that detect the displacement of the skin [1–3], accelerometers that detect motion at the electrode [4–7], strain gauges that detect skin deformation [7, 8], 2-d magneto resistive sensor components [4], electrode structures [9], contact pressure sensors [3], and skin-electrode impedance measurements [1, 6, 9–13]. The aim of these studies has been to use adaptive filtering on the measured signal to reduce the motion related signal [1, 4–8, 11] or to detect the motion and to consequently omit the motion artifact-infected signal [10].

Due to its ability to also measure the change of skin-electrode contact quality over time, skin-electrode impedance measurement has garnered more interest than the other methods.

Studies investigating skin-electrode impedance in relation to the motion artifact have resulted in various conclusions. Although some studies show that the change in impedance is not the cause of the motion artifact [10, 13], it is generally accepted that motion of the electrode causes motion artifact and also causes changes in electrode-skin impedance. Other studies, however, suggest that impedance is partly the cause of motion artifact [14]. Skin-electrode impedance has been found to be a suitable input parameter for the adaptive filtering of the motion artifact or for the removal of the affected signal component [1, 6, 10, 11]. A number of these skin-electrode impedance studies used single impedance current frequency: 13 Hz [14], 400 Hz [10], 2 kHz [13], 2.2 kHz [11], 100 kHz [9]. One study implemented a 2 kHz square wave current for the impedance measurement [6], and two others used multiple frequencies: 200 Hz and 2 kHz [12] and seven frequencies between 120 Hz and 1.8 kHz [1]. The contact impedance between skin and electrode, when tested on a skin dummy, showed a decrease with increasing force at 5 kHz impedance current frequency [15]. When measured on skin, there is an inconsistent yet present effect of applied force on the skin-electrode impedance [16]. Moreover, skin compression causes potential changes in the skin [17]. The skin impedance and skin-electrode impedance spectrographs have also been measured, albeit in the absence of motion, at frequencies between 1 Hz and 1 MHz [18], 0.5 Hz and 10 kHz [19], 30 Hz and 100 kHz [20], and 0.1 Hz and 100 kHz [21]. These measurements show the similar pattern of impedance decreasing with increasing frequency.

When considering the relationship of skin-electrode impedance to motion artifact, the intuitive idea is to use impedance measurement frequency in the frequency ranges of the biosignals of interest. This issue also arose in our previous paper where we used a injected current of 100 kHz for the impedance to study the motion artifact–a biopotential with frequency components well below 100 Hz [9]. As in our previous research and as in other studies [6, 10–13], the impedance measurement are carried out using injected currents at higher frequencies than the main frequency components of the surface potentials of interest or the motion artifact itself. Few studies used impedance measurements at low frequencies [1, 12].

In this study, we aim to investigate the relationship between different impedance measurement frequencies, the electrode motion pattern, and the resulting motion artifact. We apply programmable motion to the electrode and measure skin-electrode impedance at 24 different frequencies ranging from 25 Hz to 1 MHz. Following the impedance measurements, two motion artifact-containing biopotentials are measured under the same conditions and the mounting force applied to the electrodes is monitored using a novel system. Ten volunteers took part in the study. As far as we are aware, this study is the first of its kind and the results of the study can be used to guide further research into more suitable impedance frequencies that better relate to motion and/or motion artifact.

## Methods

In short, an electrode was mounted on the skin by a known applied force and was subjected to motion. Three consecutive impedance measurements, each done with 8 simultaneous frequencies, were carried out. Following the impedance measurements, a surface biopotential measurement was carried out under the same conditions and with the same applied motion. This was achieved by changing the connectors to the electrodes without affecting electrode location or subject positioning. An analysis of the data provided us with information on how the frequency of the injected current of the impedance measurement reflects on the relationship between skin-electrode impedance and the applied motion and between skin-electrode impedance and the motion artifact. As a reference point, these measurements also provided us with the relationship between the motion artifact and the applied motion.

**Figure 1:**
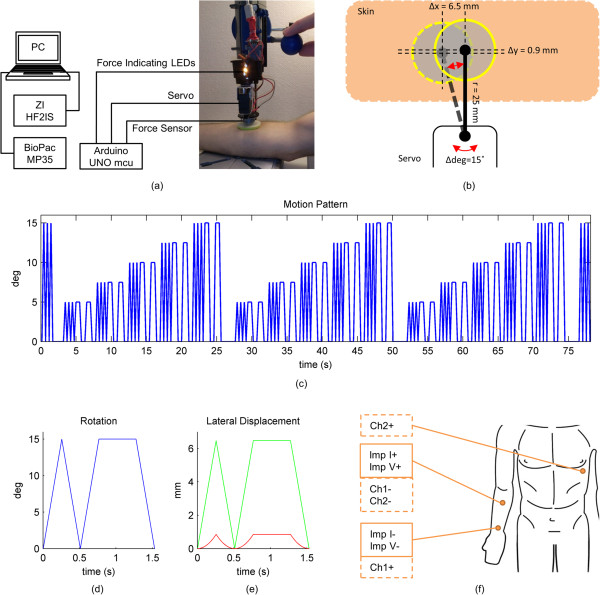
**Overview of system setup, movement generation and electrode placement. (a)** Shows the system overview with the actual motion creation module presented as pressed on the arm. The lit LED indicates the applied force is in the desired range. **(b)** Depiction of how the servo rotation is translated into lateral movement of the electrode. Depicted are a back and forth servo rotation of 15 degrees and the x- and y-axis displacements of the electrode **(c)** The rotation/motion pattern applied by the servo. **(d)** A close up of the servo rotation pattern of the largest rotation magnitude. Of the shown peaks, the second peak has a one second pause at both ends; the last pause is not shown. **(e)** The graph shows how the rotational pattern of the servo shown in Figure 1d translates into x-axis and y-axis motion. Green line is the magnitude of x-axis displacement; red line is the y-axis displacement. **(f)** The electrode setup for the impedance measurement and the biopotential recording in solid outline and dashed outline, respectively.

The electrode that was to be subjected to motion was mounted to the skin with our novel device that monitors the mounting force used to secure the electrode and applies programmed motion to the electrode. The backbone of the motion generation module was a Dremel 220 workstation (Robert Bosch Tool Corp., Illinois, USA). The vertically moving platform, originally designed to host a work tool, was modified so as to host a hexTronik HX12K Hi-speed servo (Hextronik Limited, ChenDu, China). The 25 mm extension arm of the servo was connected to a 2 part mechanism made of 3D printed plastic parts and a Kevlar tube that acted as a housing for a FlexiForce A201 (Tekscan Inc., South Boston, USA) force sensor and formed a platform with which the electrode was mounted on the skin. Figure 1a shows how the modified Dremel workstation is used to secure the electrode on the skin, as well as the location of the force sensor and the servo. An Arduino Uno microcontroller (SmartProjects, Turin, Italy) was used to program and control the servo rotation, to process the output of the force sensor, and to control the mounting force-indicating LEDs. The force sensor is a non-linear resistive sensor, and the resistive output was converted into a linear voltage reading by using an op-amp circuit, as described in the user manual of the sensor. The housing of the sensor ensured that only vertical force was transferred to the force sensor by causing the lateral movement of the servo arm to be transferred to the electrode in a way that the resulting sheer forces bypassed the force sensor. This allowed for only the pressing force to be measured and prevented or minimized the sheer forces acting on the sensor. A diagram of the main components of the system is given in Figure 1a; the electrode leads from the measurement system are not shown.

The rotational motion of the servo was translated into lateral motion of the electrode in the coordinate plane parallel to the skin, by the servo arm, as depicted in Figure 1b. Since the electrode was pressed on the skin and the used movement ranges were set so that the electrode movement was accompanied by skin stretch and deformation; no sliding of the electrode over skin was caused. As can be seen, the movement of the electrode is not a perfectly linear one axis movement. The maximum rotation magnitude of the servo arm was 15 degrees back and forth rotation and this rotation translated into 6.5 mm electrode displacement in the x-axis and 0.9 mm electrode displacement in the y-axis. The servo was programmed to the pattern shown in Figure 1c. The reason for using a triangular propagation for the rotation was practical. The servo control coding of the Arduino is simple and takes requires less execution time at the microcontroller than a more complex pattern. For studies more specific on the relationship of motion patterns to motion artifact, other patterns can be programmed. The rotation range was increased at the same ratio as the speed of movement was increased, keeping the movement frequencies constant. The frequency of the back and forth rotation without a pause is 2 Hz, and the main frequency component of the rotation with the pause at both ends is 0.65 Hz. The 2 Hz movement frequency was selected because it is similar to a motion artifact one would expect on a jogging subject, the one second pause was implemented to make the motion artifact of similar frequency with a daily walk, and to see if the impedances or the motion artifact change during this short pause. The translation of this movement to later electrode displacement of a circular nature is shown in Figure 1d and e. Figure 1d shows a close-up of the pattern in Figure 1c and e shows the corresponding electrode movement on the horizontal plane. The graphs present the largest rotation magnitude. For smaller rotation angles, the y-axis displacement will be even smaller. This motion allowed for the investigation of small electrode displacements as well as relatively large displacements, and also the effect of a small pause at the end of motion ranges. The total duration of the pattern including the starting and closing peak triplets was 77 seconds. In this paper, we have used the phrase “motion pattern” to denote both electrode movement pattern and servo rotation pattern because of the close similarity of the rotation pattern and the x-axis displacement pattern, and the comparably small amplitude of the y-axis displacement of the electrode.

As the motion was applied to the electrode, the skin-electrode interface impedance was measured as a two-electrode impedance measurement using a Zurich Instruments HF2IS Impedance Spectrometer (Zurich Instruments AG, Zurich, Switzerland). This device can simultaneously measure the real-time potentiostatic impedance at 8 frequencies. To achieve adequate resolution, the frequency band between 25 Hz and 1 MHz was divided into 24 logarithmically evenly distributed frequencies. Because the device can simultaneously measure 8 impedance channels at different frequencies, these 24 frequencies were distributed into three groups, as shown in Table 1. The three frequency groups were designed maximally interwoven so that any possible erroneous measurement could be identified. This enabled us to monitor the integrity of the measurement between the three separate measurement groups. These three frequency groups comprising eight simultaneous measurement frequencies each were then measured in consecutive measurements without altering any of the factors related to the setup. The lowest 3 frequencies were not spaced in a logarithmically even fashion because we wanted to avoid 50 Hz interference. The sampling frequency of the impedance measurement was 112 Hz per frequency channel and the measurement parameters were configured so that the impedance current for a channel stayed between 6 and 60 μA. This sampling frequency limits the measured impedance change caused by electrode movement to 56 Hz, and has not effect on the output of the measurement system relative to the impedance measurement frequencies. The two-electrode impedance measurement setup was arranged so that the electrode affected by motion was located on the right inner forearm, 5 cm distal to the wrist, and the second electrode was also located on the right inner forearm, but proximal, close to the elbow crease.

After the impedance measurements, the surface potential change at the electrode affected by the motion and an ECG channel containing this change were measured with the BioPac Data Acquisition Unit (BioPac Systems, Inc., California, USA) at a sampling rate of 200 Hz, in response to the same motion pattern. Due to safety concerns, these two devices could not be used simultaneously. The electrode setup for the surface biopotential measurements is presented in Figure 1f. The motion artifact was measured between the electrode located 5 cm distal to the wrist and the electrode located on the inner arm close to the elbow crease. This setup is minimally affected by the ECG due to not passing the heart vector and is the same as the one used in the impedance measurements. The ECG was measured between the electrode under motion and an electrode located on the V5 location on the chest.

This produced four data sets for each subject: Impedance measurement groups 1, 2, and 3, and the biopotential measurement. The data sets for a subject were synchronized using the first and last peaks of the opening and closing peak triplets shown in Figure 1c. Once they were synchronized, the data sets of initially different sample rates were resampled to the same length and sample rate.

**Table 1:** **The three frequency groupings used in the study**

Group 1	404 kHz	104 kHz	26.7 kHz	6.9 kHz	1.8 kHz	450 Hz	117 Hz	25 Hz
Group 2	636 kHz	164 kHz	42 kHz	10.8 kHz	2.8 kHz	714 Hz	184 Hz	34 Hz
Group 3	1000 kHz	257 kHz	66 kHz	17 kHz	4.4 kHz	1.1 kHz	289 Hz	74 Hz

Before each measurement, the force applied to the electrode to be subjected to motion was set to the equivalent of 750 +/- 100 grams reading on a Soehnle Siena kitchen scale with a precision of 1 gram (Leifheit AG, Nassau, Germany). This corresponded to a force of 7.35 N exerted on the electrode, and 28 mmHg pressure exerted by the electrode to skin. The 13% tolerance level of the force seems quite large, however, in the experiments we did previously we found that the motion artifact and impedance behave similarly for electrode pressures that lie between forces needed to secure the electrode on skin and forces on the electrode that cause discomfort [9], and our applied force level is in this range. This level of electrode pressure can be achieved with a tight monitoring garment made of elastic fabric [22]. The two electrodes on the forearm comprised conductive MedTex P180 (Statex Productions & Vertriebs GmbH, Bremen, Germany) silver yarn textile with a diameter of 2 cm and a 4 mm thick support pad made of Poron Impact Cushion (Rogers Corporation, Rogers, USA) material with a diameter of 5 cm. The connection of the electrodes to the electrode leads was realized by fastening the leads to male snap connectors attached to the conducting textile of the electrode structure. In cases where two leads were connected to an electrode, an adapter was constructed by soldering two male snap connectors to a female snap connector that was then snapped on the male connector of the electrode. The electrodes were moistened with four drops of tap water to simulate the presence of sweat. Sweat acts as a conductive medium between the electrodes and the skin and begins forming a few minutes after the electrode is attached to the skin [20, 23]. Other skin preparation methods such as scrubbing the topmost layer of skin or shaving the hair under the electrode were not used. The electrode at the V5 chest location was an AmBu Blu P electrode (Ambu A/S, Ballerup, Denmark).

The HF2IS impedance spectrometer measures the complex current flowing through the measured impedance. Equation (1) is used to calculate complex impedance. V_RMS_ is the root mean square of the input voltage, I_x_ is used to denote the measured real current (RMS) and I_y_ is used to denote measured imaginary current (RMS). Resistance then is the real part of the complex impedance and reactance is the imaginary part of the complex impedance. To calculate the absolute value of impedance, equation (2) was used and to calculate the phase of impedance, equation (3) was used:
123

In time domain, a visual comparison of the impedance waveforms, surface biopotentials, and the motion pattern was carried out. The baselines of the impedance waveforms for each frequency were removed using a 0.2 Hz high-pass filter (HPF) to provide the impedance variations due to motion. The DC baseline and the low frequency baseline wander were isolated by a 0.2 Hz low-pass filter (LPF). The changes related to motion were then converted into the percentage change relative to the individual baselines by dividing the amplitude of the change of the specific signal by the baseline of the said signal. In another analysis, the absolute amplitude of the change in waveforms was separately normalized between 0 and 1 for each frequency. Normalization maps the minimum amplitude of the signal waveform at hand to 0, the maximum to 1, and the values in between to the relevant value between 0 and 1.Presentation as percentage relative to the baseline, and presentation in a normalized fashion were done in order to achieve a better comparison of the motion effect. Correlation analysis between the impedance change waveforms (percent-wise and normalized), the motion artifact, and the applied motion pattern was done to assess their linear relationship.

In frequency domain, the power spectrum density (PSD) estimates of the waveforms were calculated. Because these PSD estimate amplitudes depend on the amplitudes of the waveforms, which vary greatly due to different skin-electrode impedance values at different frequencies, the PSD plots are presented in a normalized manner. This normalization between 0 and 1 allows for easier comparison by showing the ratio of energies of the different frequency components of the waveforms without the distraction of varying plot scales. In addition to visual analysis, these normalized PSDs were then compared between the impedance changes, the measured motion artifact, and the motion pattern in order to obtain the linear relationship between these waveforms in the manner of the contained frequency components.

The same calculations were done for data obtained in the absence of motion.

## Results

**Figure 2:**
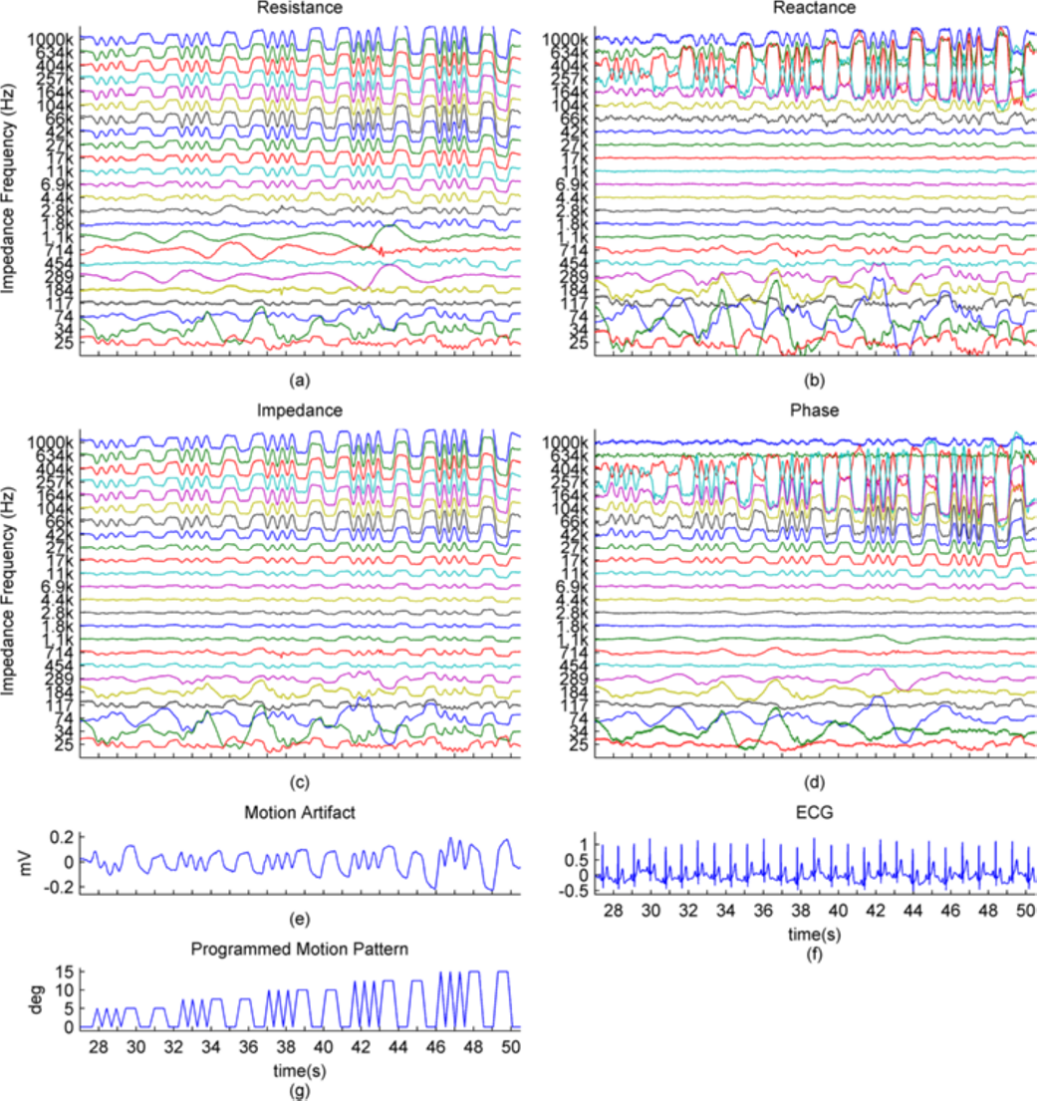
**Impedance and surface biopotential changes caused by applied motion. (a)**, **(b)**: The change of resistance and reactance in percentages relative to the baselines for each impedance current frequency. **(c)**, **(d)**: Impedance in percentages to the baseline, and the phase in degrees, for each impedance current frequency. For these four graphs, the vertical spacing between consecutive waveforms is set to correspond to a 1% change for the impedance and 1 degree for the phase. **(e)**, **(f)**: Motion artifact and ECG affected by the motion artifact. **(g)**: The motion pattern. The time window of all graphs corresponds to one run of the motion pattern, specifically the middle portion of the pattern presented in Figure 1c, around 24 seconds long. The x-axes for all graphs are same.

Sample session data from one subject, all measurements, and the calculated impedance and phase are presented in Figure 2. The percentage change of the measured resistance and reactance at 24 frequencies, the percentage change of the calculated impedance and the changes in phase, the motion artifact, and the ECG containing the motion artifact are presented. The motion pattern presented in Figure 1c has been added as Figure 2g for easier understanding. Data from this subject demonstrates the high correlation of the skin-electrode impedance to the motion pattern at higher impedance current frequencies. The presented subject is an example for subjects that have a motion artifact that correlates well with the applied motion as well as with the higher frequencies of skin-electrode impedance. The data is also an example of a subject where the lower frequencies of impedance are not well correlated with the applied motion. The effect of the motion artifact on the ECG can also be seen in the ECG plot in Figure 2f.

**Figure 3:**
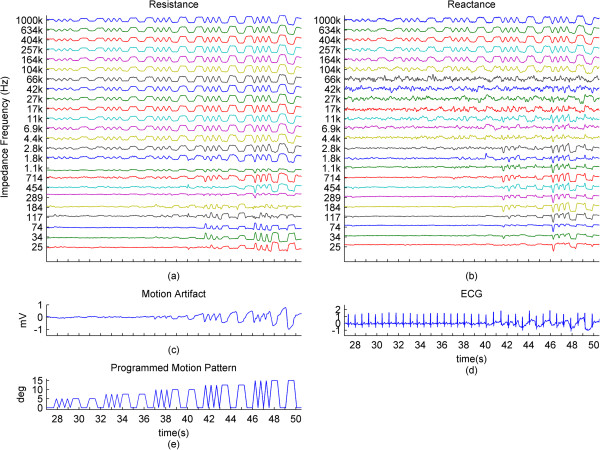
**Normalized impedance changes and biopotential changes caused by applied motion.** **(a)**, **(b)**: The resistance and reactance normalized between 0 and 1 for each impedance current frequency. **(c)**, **(d)**: The motion artifact and the ECG. **(e)**: Corresponding motion pattern. For demonstration puposes, the presented data is from a subject different than for Figure 2. This data set shows little baseline wander and good relation to motion at the lower group of impedance current frequencies. The x-axes for all graphs are same.

**Figure 4:**
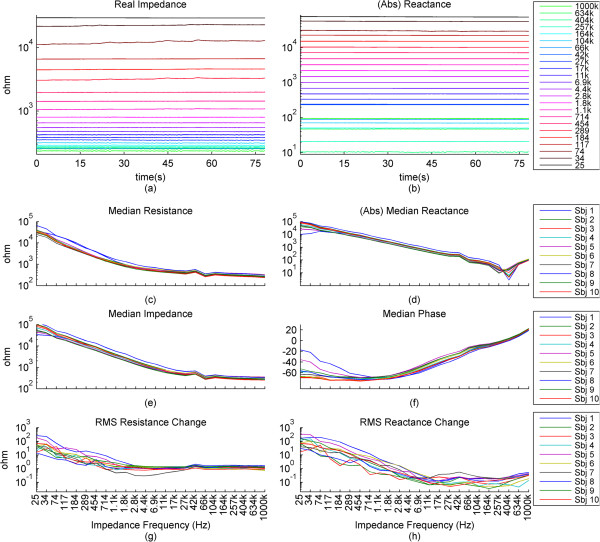
**Presenting the raw impedance signal for one subject and the median and RMS values of various impedance signals of all subjects plotted against frequency. (a)**, **(b)**: The raw resistance data and the absolute of the raw reactance data for one subject plotted against time for each impedance current frequency. **(c)**, **(d):** The medians of the resistance data and the absolute values of the reactance data for all subjects combined, plotted against impedance current frequencies. **(e)**, **(f)**: The median of the calculated impedance and of the phase of the impedance for all subjects, plotted against impedance current frequencies. **(g)**, **(h)**: The RMS values of the changes in resistance and reactance induced by motion, for all subjects, plotted against impedance current frequencies. The x-axes for the graphs **(c)**, **(d) (e)**, **(f)**, **(g)**, **(h)** are the same. All graphs except the phase are in the log scale. Thus, for the impedance values in the negative, the absolute values are presented.

**Figure 5:**
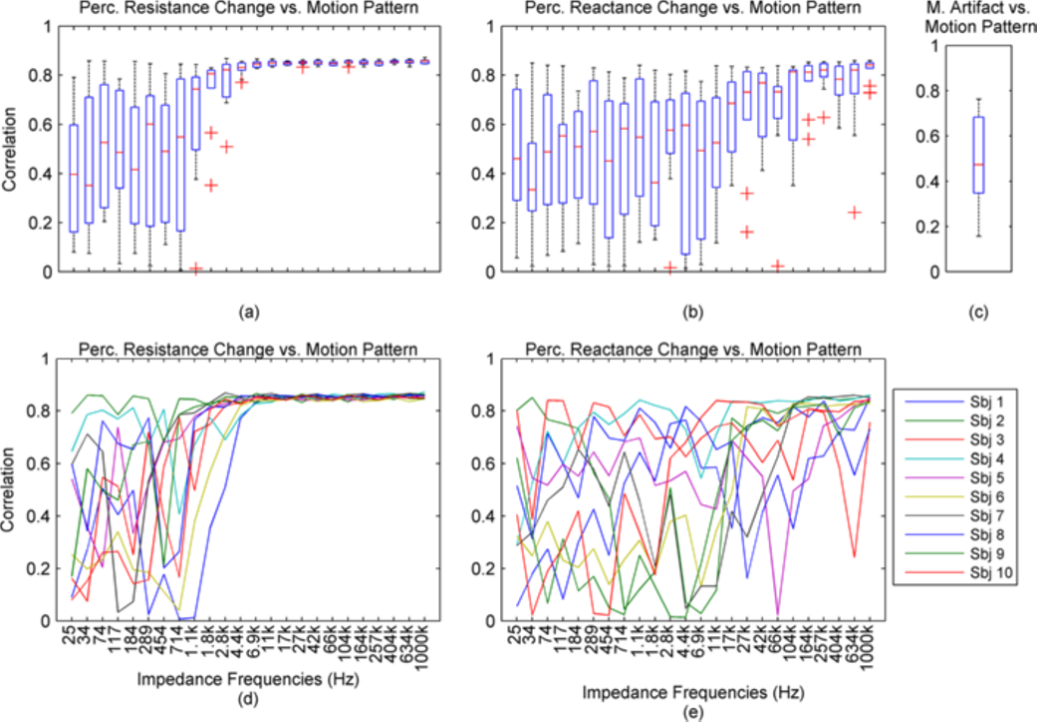
**Time doamin correlations of resistance and reactance to the applied motion pattern. (a)**, **(b)**: The boxplots of time domain correlations between resistance and the motion pattern, and between reactance and motion pattern for all subjects, plotted against impedance current frequencies. **(c)**: The boxplot of the correlation between the motion artifact and the motion pattern for all subjects. **(d)**, **(e)**: The correlation data corresponding to graphs **(a)** and **(b)**, plotted against impedance current frequencies seperately for each subject. The x-axes for all graphs are same.

**Figure 6:**
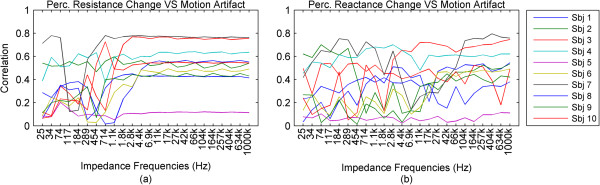
**Time domain correlations of resistance and reactance to motion artifact. (a)**: The correlations of resistance change to the motion artifact, plotted seperately for each subject against impedance current frequency. **(b)**: Thecorrelations of the reactance change to the motion artifact, plotted seperately for each subject against impedance current frequency.

**Figure 7:**
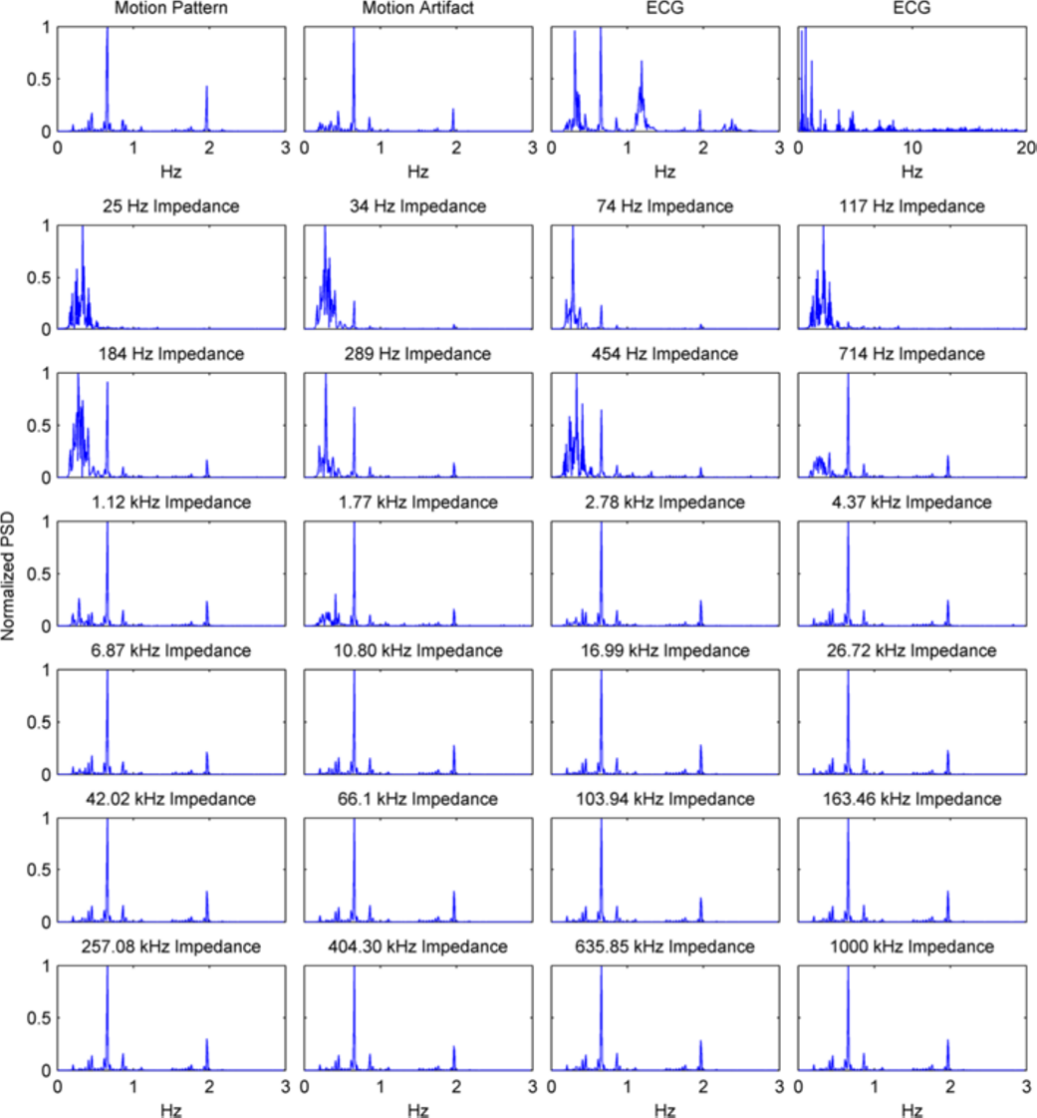
**PSDs of the motion pattern, motion artifact, ECG and impedance changes at each impedance current frequency.** On the top row, PSDs of the motion pattern, the motion artifact, and ECG are shown from 0 Hz to 3 Hz in a normalized between 0 to 1 manner. Lastly, a normalized PSD graph of ECG is shown from 0 Hz to 20 Hz for demonstration of the signal components at frequencies higher than 3 Hz. In the following rows, the normalized PSDs of the impedance measurements are shown for each impedance current frequency. The data is filtered between 0.2 Hz and 50 Hz. The x-axes for all graphs are same, except the top right corner plot.

**Figure 8:**
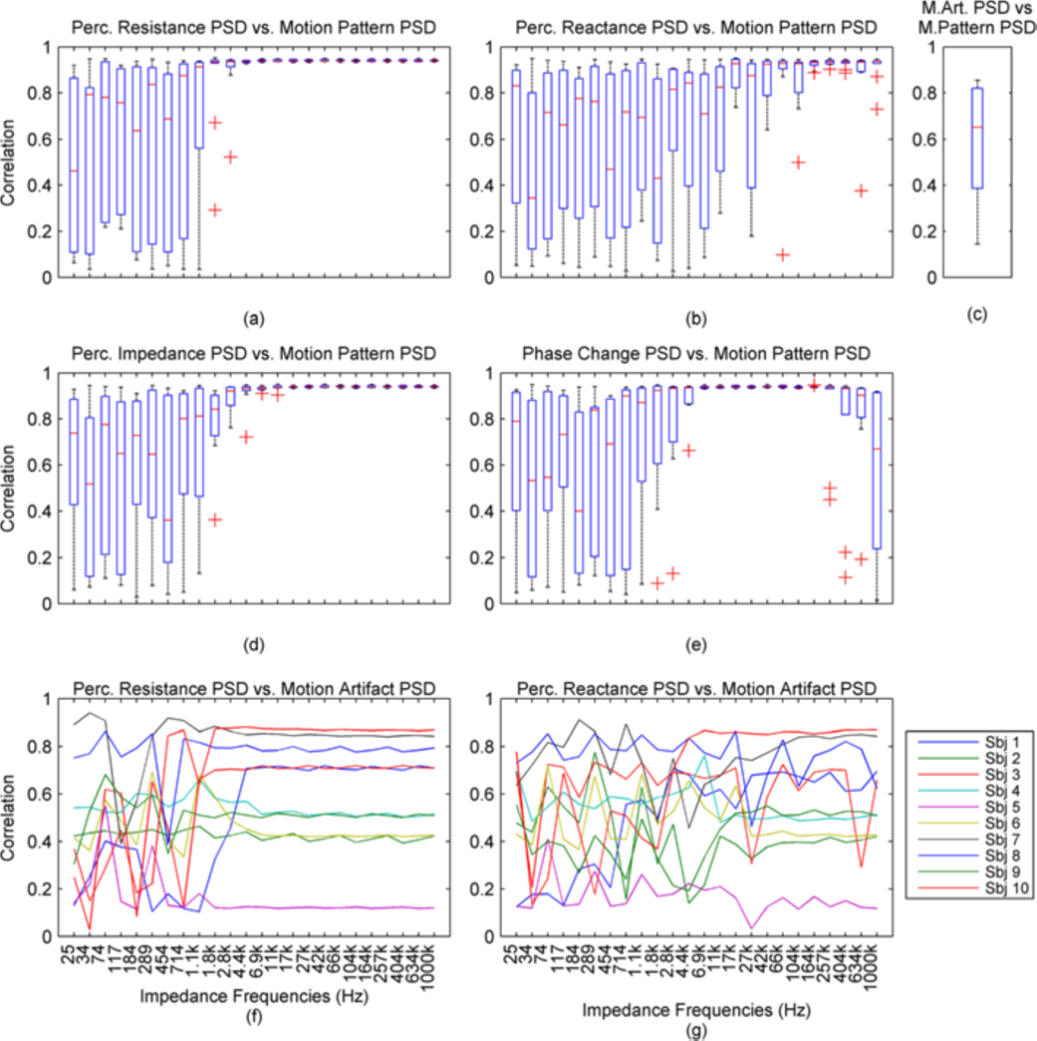
**Correlations of the PSDs of impedance signals to the motion pattern and the motion artifact. (a)**, **(b)**: The boxplots of the correlations of the PSDs of the resistance and reactance changes to the motion pattern are presented. **(c)**: The correlation of the PSD of the motion artifact to the PSD of the motion pattern. **(d)**, **(e)**: The boxplots of the correlations of the PSDs of the impedance and phase changes to the motion pattern are presented. **(f)**, **(g)**: The correlations of the PSDs of the impedance to the PSD of the motion artifact are shown seperately for each subject to demonstrate the differences between subjects. The x-axes for all plots are the same.

**Figure 9:**
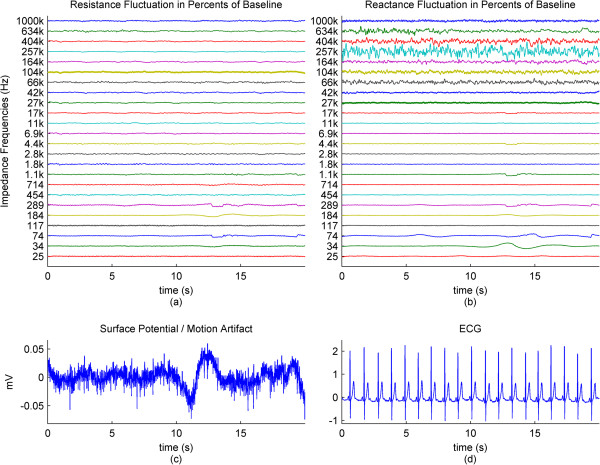
**Presented is data during steady state in the absence of motion. (a)**, **(b)**: The resistance and reactance for one subject plotted against time, for each impedance current frequency. **(c)**, **(d)** The motion artifact and the ECG. The fluctuations in the high frequencies of reactance are not observed in the resistance. Of note is the small scale of the motion artifact graph, **(c)**. The x-axes for all graphs are same.

For further results, we have omitted the calculated impedance and the phase, since they are directly derived from the resistance and reactance.In the previous graphs, we have presented the change of the impedance in percentages relative to the baseline of the impedance. In some cases where this percentage was higher in higher frequencies and lower in lower frequencies, the waveforms became hard to visually analyze due to overlap and being too small, relative to their specific impedance baselines. In Figure 3, we present the impedance change for each frequency normalized between 0 and 1. We present a subject that has good correlation of the impedance especially to the higher ranges of the motion pattern throughout the frequency range.The baselines for the resistance and reactance at the different frequencies for one subject are shown in Figure 4a and b. The medians of the resistance and reactance for all subjects in respect to impedance current frequency are shown in Figure 4c and d. In Figure 4e and f, we present the median impedance baseline and the associated phase baseline for all subjects in respect to impedance current frequency. The resistance decreases considerably for the increasing impedance measurement current frequency. The reactance starts off in higher amplitudes in the negative, gradually changes towards the positive, changing sign around 257 kHz, which is observed as an upwards bend of the reactance. In Figure 4d, plot is presented in the log scale thus the sign of the signal is not shown, only the absolute amplitude. The negative reactance and the progression into positive reactance can be also observed from the phase plot, Figure 4f. In Figure 4g and h we present the root mean square (RMS) values of the amplitudes of resistance and reactance changes induced by motion.The correlations giving the linear relationship between the time domain waveforms of the impedance changes in percentages and the motion pattern are presented in Figure 5, as a combination of all subjects. In Figure 5a and b, we present the boxplots of the correlations to give a general overview of the behavior, and then in Figure 5d and e. we present the correlations for each subject to give a more detailed idea. For a better understanding, we also present the correlation of the motion signal to the applied motion pattern as Figure 5c.The time domain correlations between the impedances and the motion artifact for each subject is shown in Figure 6. The stability of the relationship between resistance and motion artifact in the higher frequencies that deteriorates in lower frequencies can be seen in Figure 6b.The power spectrum densities for the impedances, the motion artifact, the ECG and the motion pattern are shown in Figure 7. The graphs present data that is normalized between 0 and 1 so that the maximum of the individual data is set to 1, the minimum to 0, and the rest of the data appointed a corresponding value between 0 and 1. After removing the baselines by the 0.2 Hz high pass filter, more than 99% of the signal power is contained in the frequencies between 0.2 and 3 Hz. As the remaining frequency components can be considered negligible, the plots show 0.2 Hz to 3 Hz. To demonstrate the negligible components, the PSD of the ECG from 0 Hz to 20 Hz is shown in the top right corner. The data is filtered from 0.2 Hz to 50 Hz to eliminate a low frequency drift not caused by the motion.The correlations of the normalized PSDs of the impedances at various frequencies to the programmed motion pattern were calculated to investigate the frequency domain similarity of these waveforms. The results are shown in Figure 8a,b,d and e. As a comparison, we also present the correlation of the PSD of the motion artifact to the applied motion pattern in Figure 8c. In Figure 8f and g, we present the correlations of the PSDs of the impedances to the PSD of the motion artifact seperately for each subject to demonstrate the inter-subject variability. As seen in Figure 8a and b, this inter-subject variability does not exist to the same extent for the resistance PSD and the reactance PSD correlations to the applied motion pattern PSD.To demonstrate the low frequency baseline fluctuation of the surface potential and the stability of the impedance under steady case, a 20-second long segment of measurements in the absence of motion is presented in Figure 9. The presented data comprises resistance change as a percentage of the resistance baseline amplitude (Figure 9a), reactance as a percentage of the reactance baseline value (Figure 9b), surface potential (Figure 9c), and ECG (Figure 9d). A low frequency fluctuation in the lower frequencies of resistance and a high frequency noise in the higher frequencies of reactance can be observed. The motion artifact fluctuation is very low compared to the ECG.

## Discussion

The results of previously published studies on the correlation between skin-electrode impedance and the motion artifact have varied. The studies have found that motion at the electrode location creates motion artifact and causes the skin-electrode impedance to change [1, 6, 10, 11], and that the skin-electrode impedance change is not the reason for the motion artifact [12, 13]. These studies used different frequencies of impedance measurements and it is unclear as to what extent the choice of impedance current frequencies has affected their results. Here, to the best of our knowledge, we have simultaneously estimated the motion-generated impedance changes in a large frequency band for the first time.

To investigate the effect of different impedance measurement frequencies on the relationship between skin-electrode impedance and applied motion and between the skin-electrode impedance, we measured 24 frequencies of impedance in a spectrum between 25 Hz and 1 MHz and applied a programmed motion pattern under monitored electrode mounting force. Consequently, as the electrodes were subjected to the same motion pattern, we measured the motion artifact as a surface biopotential.

In our results, we found that impedance measurements at different current frequencies have different waveforms in response to the same applied motion. In the context of motion studies, higher frequencies of impedance measurement possess a better relationship to motion than lower frequencies in a frequency range of 25 Hz to 1 MHz. Motion has been seen to cause the motion artifact, but the relationship is not as close and linear as it is between impedance and the motion pattern.

In our measurements, the skin-electrode impedance of the textile electrode for all subjects followed a similar pattern. The resistance baseline decreased with an increasing frequency. The reactance baseline, negative for the lower frequencies, decreased in absolute magnitude, until around 257 kHz where it passed into positive, and then showed a slight increase in the positive axis. This effect is consequently seen in the phase as an overall increase from a median of -60 degrees to 21 degrees. The impedance, calculated with equation (2), followed the resistance and reactance by increasing for decreasing frequencies.The amplitudes of the changes in the impedance were related to the baseline amplitudes of the impedances at the respective frequencies and thus varied greatly in absolute amplitude. For a better understanding of these changes, we decided to present the changes in percentages relative to the baseline impedances at the given frequencies. The frequencies presented in Figures 2 and 9 are set up so that the y-axis distance between each consecutive waveform corresponds to a 1% change. These results show that the applied motion can be visually detected from the impedance, resistance and reactance and phase. For example, for impedance, the visual relationship is clear for frequencies higher than 1.8 kHz, yet it exists also in lower frequencies. The reactance also clearly showed the effect of motion, but resistance had a clearer relation to the applied motion. Resistance generally showed a larger percentage change in higher frequencies than did the reactance.

PSD analysis of the resistance and reactance shows negligible signal components in frequencies higher than 2 Hz, the highest frequencies present in the motion pattern. After the removal of the baseline wander by a 0.2 Hz filter, the signal power contained between 0.2 Hz and 2 Hz was over 97% of the signal components at frequencies higher than 0.2 Hz. Between 0.2 Hz and 3 Hz, this ratio was over 99%. This shows that the change imposed on the impedance by the motion stays in the frequencies of the motion. Another observation that can be made is that while the large electrode displacements can also be easily seen in low frequency impedance measurements, in some cases the low frequency impedance seems to be less sensitive to small displacements than the higher frequency impedance measurements.To eliminate the effect of the change percentage being too small to be displayed in the percentage graph, we normalized the impedance change between 0 and 1 for each frequency. Figure 3 shows a data set for which the change is present in all the frequencies, but the change differs in amplitude and in percentage ratios relative to the specific baseline. The same figure also shows that the impedance at higher frequencies is visually more similar to the motion pattern, with less distortion. This finding is supported by our correlation analysis.

The surface potential measured between the two forearm locations, including minimal ECG, shows that the motion artifact is caused and related to the applied motion and that the ECG measured between the wrist and ECG electrode V5 location shows that the motion artifact is distortive on the ECG signal. This was reported in our earlier work [9].

The correlations of the time traces of impedances to the motion pattern clearly show this relationship. For all subjects, the correlations of the resistance to the time trace of the applied motion are larger than 0.8 for higher frequencies starting at 11 kHz. This linear relationship is not as clear between reactance and the applied motion yet also for reactance the existing relationship improves with increasing frequency. The correlations of the impedance and phase are a very similar to resistance and reach a level larger than 0.8 at 17 kHz.

In our test group, the motion artifact correlates to the programmed motion pattern beween 0.15 and 0.76, with a median of 0.5. The stable correlations between motion artifact and impedance are obtained in frequencies above 42 kHz. The median of these correlations is lower than the median of the correlations between impedance and motion pattern, and this is probably due to the same anatomical or physiological factors that cause the correlation between the motion artifact and programmed motion pattern to be lower than the correlations between impedance and motion pattern. The reason why the motion artifact seems to have a non-linear relationship to the applied motion while the skin-electrode impedance seems to have a linear relationship to the applied motion, when both are caused by the same motion and in the same section of the signal pathway, needs to be studied further.

We observed that the two subjects which have the lowest correlations have a higher body mass index (BMI) than the other subjects and that the motion artifact of these subjects is lower than the other subjects. In addition, the ECG observed between the arm electrodes was unexpectedly high for these subjects. We are not sure if and how these are related, yet an educated guess is that these two factors, when combined, might further explain the lower correlation and also be a possible subject for further studies.When looking at the PSDs normalized for ease of comparison and the correlations of the PSD’s of the impedance to the PSD of the applied motion, the above mentioned relationship is also observed. The main frequency components of the resistance change almost completely mirror the frequency components of the applied motion, with a median correlation of over 0.9 at impedance frequencies above 1.8 kHz. For impedance the median correlation is above 0.9 starting at 2.8 kHz and for reactance the median correlation is above 0.9 starting at 42 kHz, yet with a larger variability than resistance or impedance. The phase PSD has a correlation to the applied motion pattern that has a median 0.9 between 1.8 kHz and 404 kHz, yet at both ends of this range, the variation of the phase is higher than in the middle parts of this range, as seen in Figure 8e.

The low correlations of the lower frequencies of impedance to the applied motion and the even lower correlations to the motion artifact that have large inter-subject variability might be the reason why some of the previous studies have found varying relationships between impedance and motion artifact.To observe what happens when there is no motion, we included a measurement in the absence of motion. We observed some fluctuations in the impedance waveforms and the motion artifact signal, but could not draw a relationship to the motion pattern in the time or frequency domain. These changes in the skin-electrode impedance and surface biopotential in the absence of motion and stable, applied force might be due to the subject involuntarily moving while breathing, or skin conductivity or voltage changes related to blood flow. However, the changes observed are very small and can be seen from the surface potential plot in Figure 9. The surface potential has a 0.1 mV peak-to-peak value in the form of a baseline drift, and thus it does not considerably affect motion artifact measurements. Figure 9 also shows, by proxy of impedance and motion artifact, that when the electrode is pressed on the skin and motion is absent, the mounting force applied to the electrode remains steady.

In terms of accuracy, repeatability and the effect of using a +/- 100 gram range for force levels, the differing electrical skin properties between subjects and also for the same subjects at different times cause larger differences than can be expected from the force range or system specifications. The BioPac measurement system has a resolution of 3 μV, the accuracy of the HF2IS is given as 1% for the output and max 5% at 5 MHz for the input and the force sensor has a linearity error of max +/- 3% and a repeatability error of +/-2.5%. In the context of electrode movement, especially in locations with bone and tendons close to the electrode location, small electrode location changes of even a few millimeters can have a more drastic effect on the motion artifact than caused by the inaccurancies due to the system components. Thus, we conclude that even our the system has some uncertainity due to the devices and experiment protocol, more important is electrode location selection and location standardization, and the testing of a high number of subjects. As we are measuring impedance in relation to motion and that the changes in impedance are more meaningful than absolute impedance, concluding from the similar trend for the data from our subjects, we think that the tolerance and accuracy levels of the system and protocols are adequate.

## Conclusion

To the best of our knowledge, this is the first paper in which the relationship between electrode motion and motion artifact biopotential to the impedance changes of the electrode over a wide frequency scale of continuous impedance measurements during the motion is studied. We observe that the baseline levels of skin-electrode impedances are similar to those observed by other studies: higher impedance at lower frequencies. The peak-to-peak amplitude of the impedance change initiated by the motion also follows the same pattern. The relative percentage change in the impedance is in the range below 2% for most of the frequencies and measurements.

The important observation in our study is that all frequencies are not equal in electrode impedance measurement during motion. Furthermore, if the aim is to use the impedance change to detect or reduce the motion artifact, the best approach is not to use the frequencies associated with standard biosignal, ECG, and EMG that are below 500 Hz, but instead to use impedance measurement frequencies at ranges that correlate best with motion. For impedance, regarding our selected impedance measurement frequencies, this range lies between 17 kHz to 1 MHz, and for resistance it lies between 11 kHz to 1 MHz, yet for reactance a good linear time domain correlation was not present. Another advantage of using higher frequencies for impedance measurement is that the impedance measured at these current frequencies seems to be more sensitive to small movements and gives more accurate assessments of the applied motion at all motion levels.We observed that the motion applied causes motion artifact. However, the motion artifact shows lower correlation to the applied motion than the impedance. This correlation has a large variation between subjects, as seen in Figure 6 and Figure 8f and g. It can also be seen that the higher frequencies of impedance could be used as a proxy to applied motion. Thus, while the impedance cannot directly be used as a predictor of the motion artifact signal shape in time domain, it can be used as a predictor of the main frequency components of the motion artifact and it is an excellent predictor of the applied motion itself.

The idea that the lower frequencies of impedance measurement could be better correlated to the motion artifact was not supported by our findings where in most cases the correlation in lower frequencies of impedance measurement to motion artifact was lower and the difference between frequencies was much higher than in the higher frequencies. Even with lower correlation coefficients, the higher frequencies posessed stability in their relationship to the motion artifact. It is important to note, however, that these findings might only be valid for textile contact electrodes with a flat contact surface.

To conclude, according to our results from textile electrodes, the best way to use skin-electrode impedance to detect motion or to use it as a predictor in adaptive filtering to remove motion artifact is the use of frequencies higher than 17 kHz in the case of impedance where both the time domain signals and the frequency components respectively present a correlation higher than 0.8 and 0.9 to the applied motion. This relationship held true up to 1 Mhz, the highest frequency in our experiments. If resistance is to be used, then the frequencies higher than 11 kHz provide the best relationship to the applied motion.
